# Editorial: Immunity and inflammation in neuropsychiatric disorders: pathogenesis, detection methods, novel therapeutic approaches, perioperative management, and anesthetic influences

**DOI:** 10.3389/fneur.2025.1609898

**Published:** 2025-05-13

**Authors:** Zhanwei Hu, Jie Zeng, Cong Yu

**Affiliations:** ^1^Department of Anesthesiology, Stomatological Hospital of Chongqing Medical University, Chongqing, China; ^2^Chongqing Key Laboratory of Oral Diseases and Biomedical Sciences, Chongqing, China; ^3^Chongqing Municipal Key Laboratory of Oral Biomedical Engineering of Higher Education, Chongqing, China

**Keywords:** neuropsychiatric disorders, immunomodulatory approaches, anesthetic implications, neuroinflammation, peri-operative management, neurological disease detection methods

Neuropsychiatric disorders, with their current high prevalence, unclear etiology and poor treatment, remain a major health problem for the world. To enhance our understanding of the clinical manifestations and underlying mechanisms, we have accepted eight studies under the research theme of “*Immunity and Inflammation in Neuropsychiatric Disorders*”. Two network meta-analyses of psychiatric disorders, five studies on immuno-inflammation, narcotics on CNS genesis, treatment, and prevention, and one review were included. These studies also explored new biomarkers and therapeutic strategies, emphasized the translational potential of immunomodulatory approaches in neuropsychiatry, and addressed neuropsychiatric disorders from multiple perspectives including the understanding of immune cells, inflammatory biomarkers, and imaging., inflammatory biomarkers as well as imaging alterations, general anesthetic medications and perioperative protocols.

In this review, Faustmann et al. systematically explored the key contribution of microglia in schizophrenia, which may be involved in the disease process through mechanisms such as pro-inflammatory factor release and abnormal synaptic pruning (1). The author innovatively proposed an astrocyte—microglia co—culture model (M5/M30) in the study, which can simulate physiological and pathological state (2). The model may deepen the understanding of drug mechanisms of action and facilitate the development of novel therapies targeting glial dysfunction, while improving both positive and negative symptoms in patients. Further support for the “microglia promote neuroinflammation hypothesis” in schizophrenia (3).

An original study by Melamed et al. found that intravenous immunoglobulin (IVIG) significantly reduced pro-inflammatory monocyte levels and improved obsessive-compulsive disorder and tic symptoms in patients with PANS (Pediatric Acute Onset Neuropsychiatric Syndrome). This finding supports the immunoinflammatory hypothesis of PANS (4), and suggests that innate immune abnormalities may mediate neuroinflammation through monocyte/dendritic cell activation. IVIG demonstrates therapeutic potential through multi-targeted modulation (e.g., neutralization of autoantibodies, inhibition of complement activation) and offers a new way of thinking about neuropsychiatric disorders in the post-infectious setting (5).

Han et al.'s study introduces the System Immune Inflammation Index (SII) as a cost-effective and convenient novel inflammatory marker (6). The authors included 206 maintenance hemodialysis patients. High SII was confirmed to be an independent risk factor for depression. It can predict the risk of depression in hemodialysis patients. The authors concluded that SII is an ideal inflammatory marker for diagnosis and prediction of depressive symptoms in MHD patients, providing a cost-effective tool for early clinical detection.

Zhang et al.'s study examined the effects of inhalational sevoflurane general anesthesia singly on intelligence in preschool children. Following a 48-month follow-up of 232 preschoolers treated with a single dose of sevoflurane anesthesia after dental treatment, the authors found no findings that a single dose of sevoflurane impaired neurocognitive development in preschoolers (7), which gave clinicians more confidence in its safety, and that the children benefited from improved developmental malnutrition after the treatment.

Li et al. Preoperative rehydration guided by ultrasound measurement of carotid corrected flow time (FTc) was useful in preventing hypotension after general anesthesia for gastrointestinal surgery.Decrease in blood pressure after anesthesia is a common complication of general anesthesia, and prolonged hypotension affects perioperative cognitive function. The authors concluded that FTc = 340.7 ms is a critical value that can guide prehydration to combat hypotension after general anesthesia, and the particular feature of this study is the use of FTc as a simple, noninvasive tool for assessing blood volume in patients who are spontaneously breathing (8).

Yu et al. conducted a network meta-analysis to elucidate the immune cells involved in autism pathogenesis. The pathogenesis of autism is not fully elucidated and specific therapeutic agents are lacking. The authors analyzed and investigated the causal effect of 731 immune cell phenotypes on genetic susceptibility to autism spectrum disorder (ASD). Thirteen immune cell phenotypes associated with increased susceptibility to ASD were obtained by Mendelian randomization analysis. The authors concluded that the importance of CD8^+^ T-cell cytotoxicity and Treg dysfunction in mediating neuroinflammation provides new targets for ASD treatment (9, 10).

Xia et al. focused on adolescent first-episode major depressive disorder (MDD) and explored the association between adverse life events, altered brain function, and depression. Adolescence is a critical period of psychological growth and vulnerability to negative events (11). By utilizing resting-state functional MRI (rs-fMRI), the authors found abnormal activity in the right insula of adolescent depression patients, which was associated with childhood trauma and negative life events. The authors concluded that abnormal ROL function may be a potential biomarker for depression and emphasized the impact of environmental stress on brain function, with early life stress affecting brain development through neuroinflammation and increasing the risk of depression (12).

In conclusion, this Research Topic, “*Immunity and Inflammation in Neuropsychiatric Diseases*,” provides an overview of the mechanisms of neuropsychiatric disease pathogenesis and progression, as well as an understanding of the impact of general anesthesia on intraoperative management and postoperative regression from a perioperative perspective. The studies discussed emphasize the work needed to implement effective interventions and prevention strategies, and the variety of new management approaches and medications highlights the importance of research. We also endeavor to provide more opportunities for researchers to present their works.
